# Multi-omics analysis reveals ThMYB6 regulation of flavonoid biosynthesis in differently colored tuberous roots of *Tetrastigma hemsleyanum*

**DOI:** 10.3389/fpls.2025.1642835

**Published:** 2025-09-08

**Authors:** Feng Yang, Qiaoyue Xing, Shouzan Liu, Yiwen Gu, Ziyue Wang, Hongyan Wang, Qiong Shen, Shan Li, Bojie Feng, Yan Bai

**Affiliations:** ^1^ National Key Laboratory for Development and Utilization of Forest Food Resources, Zhejiang Agriculture and Forestry University, Hangzhou, Zhejiang, China; ^2^ College of Food and Health, Zhejiang A&F University, Hangzhou, Zhejiang, China; ^3^ Botanical Garden, Zhejiang A&F University, Hangzhou, Zhejiang, China; ^4^ General Station of Zhejiang Forestry Extension, Hangzhou, Zhejiang, China

**Keywords:** *Tetrastigma hemsleyanum*, metabolome, transcriptome, flavonoids, MYB factor, molecular mechanism

## Abstract

**Introduction:**

*Tetrastigma hemsleyanum* Diels et Gilg (Vitaceae) is a medicinally valuable plant typically with a whitish root cross-section. Resource surveys discovered some roots of *T. hemsleyanum* with a yellow-brown root cross-section and stronger antioxidant properties. However, the molecular mechanisms underlying these phenotypic differences remain poorly understood.

**Methods:**

We employed integrated metabolomic and transcriptomic analyses to compare the two root types. Key candidate genes were cloned and functionally characterized to uncover the regulatory mechanisms involved.

**Results:**

Metabolomic analysis identified significant changes in 217 metabolites between the two types, among which 57 flavonoids, such as Hesperetin-7-O-glucoside, were significantly higher in the yellow-brown roots. Transcriptomic analysis revealed 3516 differentially expressed genes, many associated with flavonoid biosynthesis. Differences in root quality were largely attributed to varied flavonoid production. Analysis of transcription factors regulating this process identified a strong correlation between *MYB6* genes and flavonoids biosynthesis. Functional analysis indicated that the ThMYB6 factor of the R2R3 MYB subgroup 5 regulates flavonoid production in *T. hemsleyanum*.

**Conclusion:**

The ThMYB6-mediated divergence in flavonoid biosynthesis is the key factor influencing quality differences between the two phenotypes. These results establish a theoretical foundation for targeted quality improvement and offer scientific support for the medicinal development of *T. hemsleyanum*.

## Introduction

*Tetrastigma hemsleyanum* Diels et Gilg is a perennial herb with tuberous roots that offer medicinal values, commonly called ‘Sanyeqing’. Its biologically active substances include flavonoids, phenolic acids, polysaccharides, terpenoids, and alkaloids (Wang et al., 2018). *T. hemsleyanum* is known as a natural plant antibiotic due to its powerful pharmacological activities, such as anti-inflammatory (Wang et al., 2022), antioxidant (Shu et al., 2023), antiviral (Feng et al., 2024), and antitumor effects (Liu et al., 2018). As a medicinal plant endemic to China, *T. hemsleyanum* is primarily found in Zhejiang, Fujian, Jiangxi, Hunan, Guangxi, Guizhou, and Yunnan Provinces. Phylogeographic studies divided it into two lineages: the Southwest one and the Central-South-East one (Wang et al., 2015). Currently, *T. hemsleyanum* has been designated as one of the authentic medicinal materials in the “New Eight Flavors of Zhejiang” by Zhejiang Province, with cultivation distributed throughout the entire province. Nevertheless, variations in habitat conditions and cultivation practices across different producing regions have led to disparities in the quality of *T. hemsleyanum* medicinal materials (Chen et al., 2022a; Xia et al., 2023). In our resource surveys, two types of tuberous roots with distinct cross-sectional colors have been identified in the same cultivation base.

Color can serve as an indicator of herb quality and pharmacological effects. The biosynthesis and accumulation of plant secondary metabolites, like carotenoids, flavonoids, and betalains, decisively influence the color of plant tissues (Xu et al., 2023). For instance, *Lonicera japonica*, an important antiviral medicinal plant, is known as golden-and-silver honeysuckle because of the critical role of β-carotene in its transition from white to gold flower (Pu et al., 2020). Research found that the color of licorice roots is highly significantly correlated with the contents of liquiritin, isoliquiritin, isoliquiritigenin, ammonium glycyrrhizinate, and glycyrrhetinic acid. Liquorice roots with a yellower color often have higher contents of active ingredients (Ma et al., 2017). The red *Salvia miltiorrhiza* contains higher amounts of tanshinone and dihydrotanshinone, which color the root epidermis (Su et al., 2021). The yellow *Chrysanthemum morifolium* offers higher amounts of phenylpropanoids and flavonoids than the white chrysanthemum, potentially explaining the color variances (Zou et al., 2021).

Our previous research found some roots of *T. hemsleyanum* have yellow-brown root cross-sections in Linhai, Zhejiang Province, different from the common whitish cross-section tuberous roots. Studying the quality difference of the two types can contribute to the quality stability of *T. hemsleyanum*. However, little research has been conducted on the *T. hemsleyanum* with yellow-brown root cross-sections. This study compared the quality differences of the two *T. hemsleyanum* types, found that the yellow-brown roots have stronger antioxidant activity and richer flavonoid content. Furthermore, the molecular mechanisms underlying those differences were illustrated via metabolomic and transcriptomic analyses. The findings could promote the development and utilization of *T. hemsleyanum* resources and provide a theoretical basis for cultivating high-quality *T. hemsleyanum*.

## Materials and methods

### Plant materials

Three-year-old *T. hemsleyanum* tuberous roots were harvested from the cultivation bases in Linhai, Taizhou, Zhejiang Province, which were identified as *T. hemsleyanum* by Dr. Bai Yan of Zhejiang Agriculture and Forestry University. Based on the cross-section color, the roots were categorized into yellow-brown (Br) and whitish (Wh) groups. More than 5 tuberous roots were used as a biological replicate, and 3 biological replicates were collected for each color type. All samples were frozen and stored at –80°C for subsequent experiments.

### Color analysis

The tuberous root color was measured using a CR - 10 Plus colorimeter (Konica Minolta, Singapore) with a D65 illuminant at an observer angle of 10° and a window diameter of 8 mm. The International Commission on Illumination (CIE) parameters of brightness (L*), redness-greenness (a*), and yellowness-blueness (b*) were measured, and the color difference was assessed based on total aberrations (Δ*E*). In the case of uneven root cross-section color, two locations were selected to measure the color.

### Determination of antioxidant capacity

After freeze-drying, the root samples were ground into powder. Then, 0.1 g powder was subjected to extraction using 25 mL of 75% ethanol solution in an ultrasonication bath (400 W) at 55°C for 80 min. The extract was filtered before determination of antioxidant capacity by FRAP and DPPH assays. Following the manufacturer’s instructions, the FRAP assay was performed using the T-AOC determination kit (Jiancheng, China). Briefly, 5 μL extract was mixed with 180 μL FRAP buffer, and the mixture was incubated at 37°C for 4 min. The optical density (OD) was measured at a wavelength of 593 nm. The FRAP antioxidant capacity was calculated using FeSO_4_ as the standard. The DPPH assay was performed according to previous methods (Xiao et al., 2022). Briefly, sample solutions (0.2 mL) were added to the DPPH solution (0.04 mg/mL, 1.8 mL), and the mixtures were incubated at 37°C for 30 min in the dark. Finally, the absorbance of the mixtures was detected at 517 nm. Methanol (0.2 mL) mixed with DPPH solution (0.04 mg/mL, 1.8 mL) served as the control. All experiments were performed in triplicate, and the results were expressed as IC50.

### Quantitative determination of total phenolics, total flavonoids, total proanthocyanidins, and total anthocyanins

The ethanol extract was also used to determine the total phenolic contents (TPC), total flavonoids contents (TFC), and total proanthocyanidin contents (TPAC) according to the methods in the literature (Bai et al., 2022). The total anthocyanidin contents (TAC) were measured according to the manual of the Anthocyanidin Detection Kit (Yuanye, China). In brief, 0.25 g of fresh sample was thoroughly ground in a precooled mortar with 3 mL Anthocyanidin Assay Buffer. The homogenate was transferred into a centrifuge tube, and the volume was supplemented to 10 mL with the anthocyanidin assay buffer. After settling in the dark for 20 min at 4°C, the mixture was centrifuged at 8000 rpm for 3 min. The supernatant was collected for absorbance detection at 530 nm.

### Wide-target metabolomic analysis

Briefly, 50 mg of root powder was subjected to extraction using 700 μL of 75% methanol solution (containing the internal standard) at 4°C overnight on a shaker. After centrifugation at 12,000 rpm for 15 min, the supernatant was filtered through a 0.22 μm microporous membrane for further UPLC-MS analysis. In the meantime, 20 μL of the filtrate of each sample was mixed as a quality control (QC) for repetitive evaluation. The UPLC analysis was conducted using an EXION LC System (SCIEX) equipped with a Waters Acquity UPLC HSS T3 column (1.8 μm, 2.1 × 100 mm) under a flow rate of 0.4 mL/min, a column temperature of 40°C, an autosampler temperature of 4°C, and an injection volume of 2 μL, with eluent A being 0.1% formic acid-water and eluent B being acetonitrile. The gradient elution conditions were 0 min 98% A, 0.5 min 98% A, 10 min 50% A, 11 min 5% A, 13 min 5% A, 13.1 min 98% A, and 15 min 98% A. A SCIEX 6500 QTrap+ mass spectrometer with an IonDrive Turbo V ESI ion source was employed for MS analysis. Typical ion source parameters were set as follows: the ion spray voltage was +5500/–4500 V, the curtain gas was 35 psi, the temperature was 400°C, the ion source gas 1 was 60 psi, the ion source gas 2 was 60 psi, and the declustering potential (DP) was ±100 V. Multiple reaction monitoring (MRM) data acquisition and processing were carried out in the SCIEX Analyst Work Station (Version 1.6.3). Principal component analysis (PCA), hierarchical cluster analysis (HCA), and orthogonal partial least squares-discriminant analysis (OPLS-DA) were performed using R. Significantly changed metabolites (SCMs) were further screened with the criteria of Variable importance in projection (VIP) ≥ 1 and |log2FoldChange| ≥ 1.

### Targeted metabolomic analysis of representative flavonoids

Representative flavonoids in the methanol extracts of tuberous roots were quantified via UPLC-MS. The conditions for UPLC analysis were set as follows: the chromatographic column was a Waters ACQUITY UPLC BEH C18 column, the column temperature was 40°C, the eluent A was 0.1% formic acid-water, the eluent B was 0.1% formic acid-acetonitrile, the flow rate was 0.6 mL/min, and the injection volume was 1 μL. The gradient elution conditions were 0 min 5% B, 1 min 25% B, 3.5 min 40% B, and 4.5 min 60% A. The conditions for MS analysis were similar to those of the wide-target metabolomic analysis, with some changes. The ionization temperature was 550°C, and other optimal conditions are shown in **Supplementary Table S1**.

### RNA sequencing and transcriptomic analysis

The total RNA was isolated from the tuberous roots using the TRK1001 Total RNA Purification Kit (LC Science, USA). Following the quantity and quality analyses, the high-quality RNA was constructed into a cDNA library using the mRNASeq sample preparation kit (Illumina, USA). Then, transcriptome sequencing was performed on the Illumina Novaseq™ 6000 platform (Illumina, USA). The RNA-seq clean data had been deposited to the NCBI databases under accession number PRJNA1306786. After the *de novo* assembly, the unigenes were obtained and aligned against the Nr, GO, SwissProt, KEGG, and eggNOG databases for function annotation. The transcription factors were identified by searching against PlantTFDB. The expression levels of unigenes were indicated by the TPM (Transcripts Per Kilobase per Million mapped reads) value. The differentially expressed genes (DEGs) were identified with parameters of |log_2_FoldChange| > 1 and pvalue< 0.05 using the R package edgeR.

### Real-time quantitative PCR

The RNA of each sample was synthesized into cDNA using the HiScript II 1st Strand cDNA Synthesis Kit. qPCR was performed using the TB Green^®^ *Premix Ex Taq*™ (Tli RNaseH Plus) (Takara, China) in the CFX96 Real-Time PCR Detection System (Bio-Rad, USA). Finally, the relative expression level of each gene was calculated using the 2^−ΔΔCT^ method. The primers used are provided in **Supplementary Table S2**.

### Comprehensive analysis of metabolome and transcriptome

The SCFs, differentially expressed flavonoids biosynthesis genes (DEFGs), and differentially expressed transcription factor genes (DETFs) were subjected to correlation analysis on the Metware Cloud platform (https://cloud.metware.cn) with the standards of |PCC| > 0.8 and *P* < 0.05. The correlation networks between target metabolites, enzyme genes, and TF genes were plotted in Cytoscape.

### Isolation of ThMYB6 and sequence analysis

After reverse transcription, the CDS sequences of the ThMYB6 genes were amplified and cloned into the pCE2 TA/Blunt-Zero vector (Vazyme, China). Then, the ThMYB6 sequences were searched against the NCBI GenBank to identify similar genes. The protein sequences of ThMYB6 and other MYB factors regulating flavonoid biosynthesis were used to construct a Neighbor-Joining tree for phylogenetic analysis with 1000 bootstrap replicates in MEGA-X. The MUSCL plug-in of MEGA-X was used to perform multiple sequence alignments on the protein sequences of ThMYB6 and other SG5 R2R3 MYB, whose results were beautified in Gene-Doc.

### Transient expression of ThMYB6

The full-length ORF of the ThMYB6 gene was cloned into the pGreenII 62-SK vector and transformed into the leaves of *T. hemsleyanum* by *A. tumefaciens* GV3101 (pSoup). After cultivating for 2 days, the leaves were collected for analyzing the expression levels of flavonoid biosynthesis genes by qPCR. Leaves cultured for 10 days were freeze-dried and crushed to determine the total flavonoids contents. Approximately 5 g of fresh leaves were used as a biological replicate, and 3 biological replicates were collected for each group.

### Statistical analysis

All experiments were conducted with at least 3 biological replicates. The differences between means were tested for significance at the *P* ≤ 0.05 level using Student’s test in Graphpad Prism 8 (Graphpad, USA).

## Results

### Tuberous root color variation between the two *T. hemsleyanum* types

**Figure 1:**
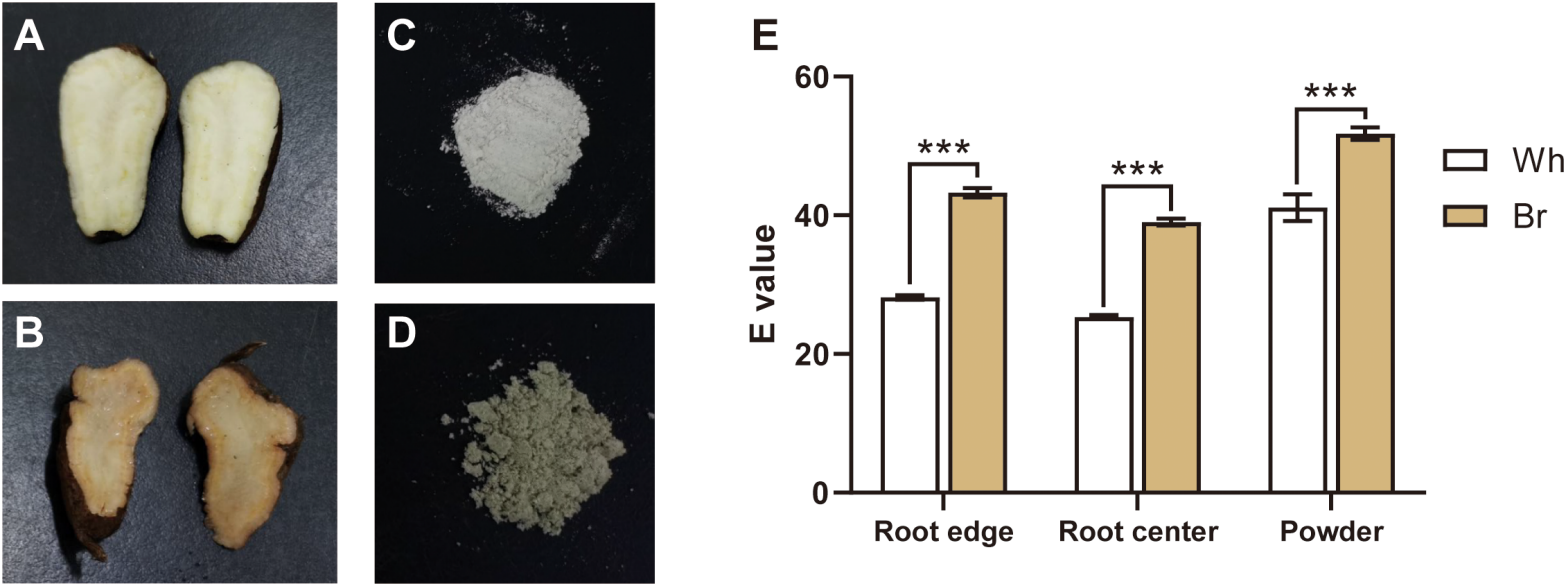
Tuberous root color comparison of the two *T. hemsleyanum* types. **(A, B)** Tuberous root cross-sections with whitish (Wh) and yellow-brown (Br) colors, respectively. **(C, D)** The lyophilized powder of tuberous roots with the whitish and yellow-brown colors, respectively. **(E)** The root color differences detected between the two *T. hemsleyanum* types, *** indicates a significant difference at *P* < 0.001.

The tuberous root cross-sections of *T. hemsleyanum* generally exhibited a whitish color (**Figure 1A**), while several were found to present a yellow-brown color (**Figure 1B**). Meanwhile, the lyophilized powder of the yellow-brown roots showed a darker color than that of the whitish roots (**Figures 1C, D**). The results of chromatic aberration analysis also showed significant color differences among the edge, center, and powder of the two types of roots (**Figure 1E**). These observations highlighted the color diversity of *T. hemsleyanum* tuberous roots and indicated their potential functional and application value differences.

### Different antioxidant capacities of the two tuberous roots

**Figure 2:**
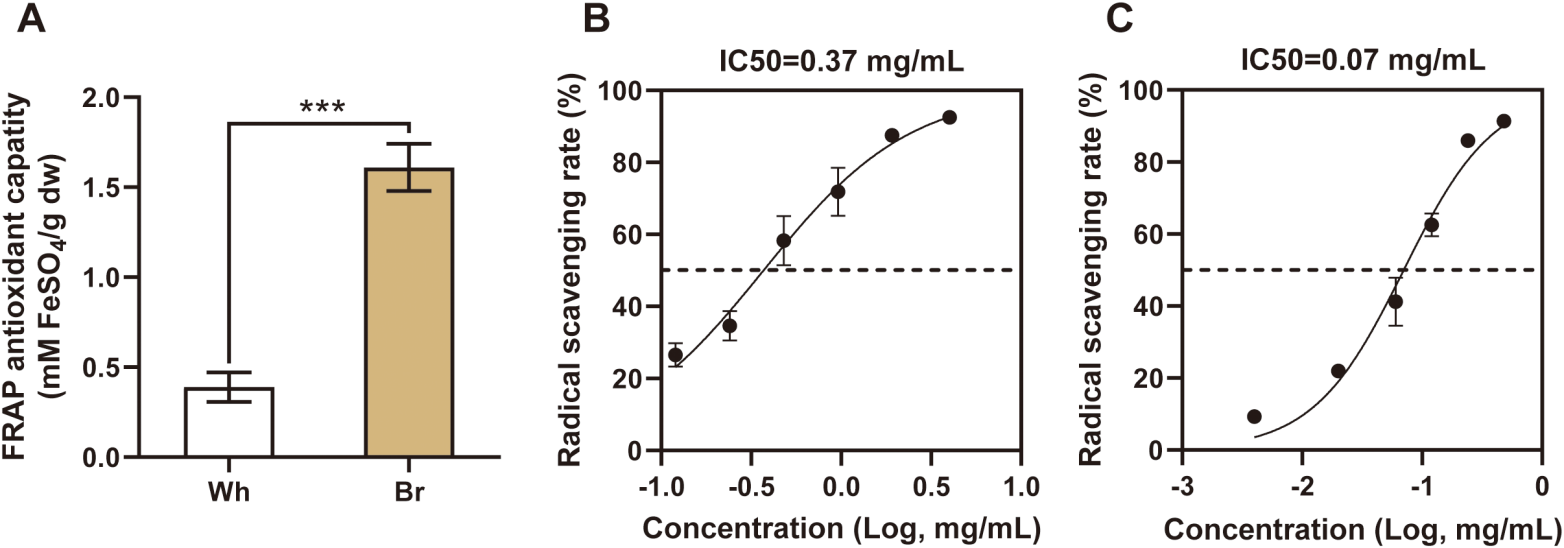
The antioxidant capacities of the two *T. hemsleyanu*m roots. **(A)** Different FRAP antioxidant capacities of the two types of roots, *** indicates a significant difference at *P* < 0.001. **(B, C)** DPPH radical scavenging rates of whitish and yellow-brown roots, respectively.

In addition to phenotypic divergences, this study revealed significant differences in the medicinal activities of the two tuberous roots. These findings provide a new perspective to explore the potential applications of different types of tuberous roots. As shown in **Figure 2A**, the FRAP antioxidant capacity of yellow-brown roots was three times that of whitish roots. In the DPPH radical scavenging assay, the IC50 value for yellow-brown roots was 0.07 mg/mL, significantly lower than that of the whitish roots (**Figures 2B, C**). These results further confirmed the greater ability of the yellow-brown roots to scavenge free radicals and prevent oxidative damage.

### Different contents of active ingredients in the two tuberous roots

**Figure 3:**
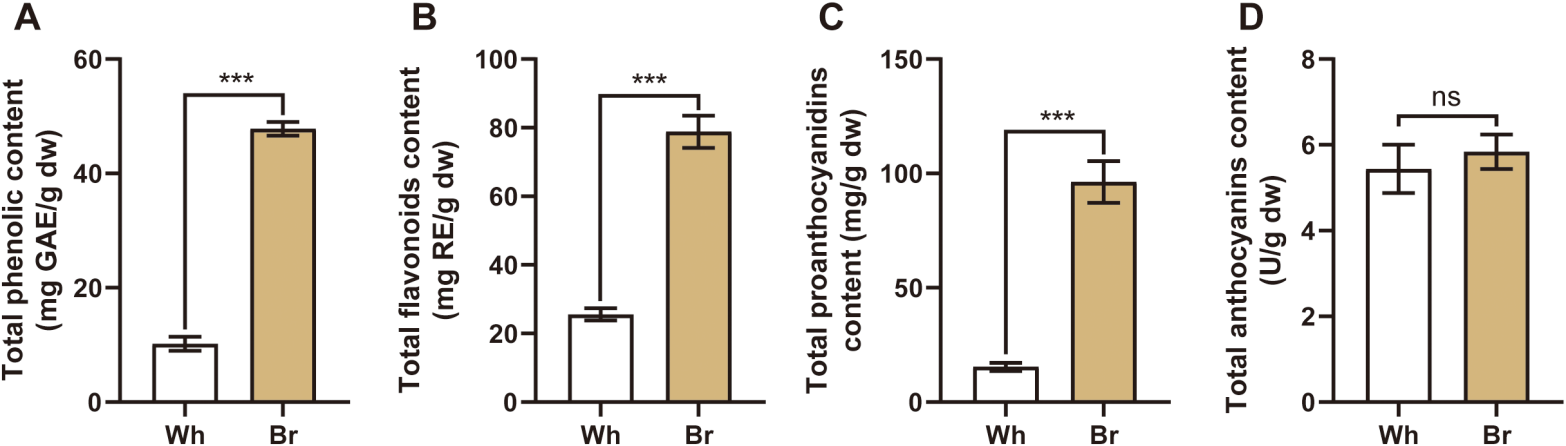
The contents of the main active substances in the tuberous roots of the two *T. hemsleyanum* types. **(A-D)** The total phenols, total flavonoids, total proanthocyanidins, and total anthocyanidins in the two tuberous roots, *** indicates a significant difference at *P* < 0.001, and ns indicates no significant difference.

This study focused on the flavonoids, the main active metabolites in *T. hemsleyanum*. The related flavonoid indexes were determined, including the total phenols, total flavonoids, total proanthocyanidins, and total anthocyanidins. The results (**Figure 3**) showed that yellow-brown roots contained significantly higher levels of total phenols, total flavonoids, and total proanthocyanidins (47.8 mg/g, 78.8 mg/g, and 96.3 mg/g, respectively) than the whitish roots. However, the total anthocyanidins in the two roots were about 5.5 U/g, with no significant differences, suggesting that anthocyanidins might not be the main components affecting the medicinal activities of *T. hemsleyanum*.

### Identification of differential flavonoids between the whitish and yellow-brown roots

**Figure 4:**
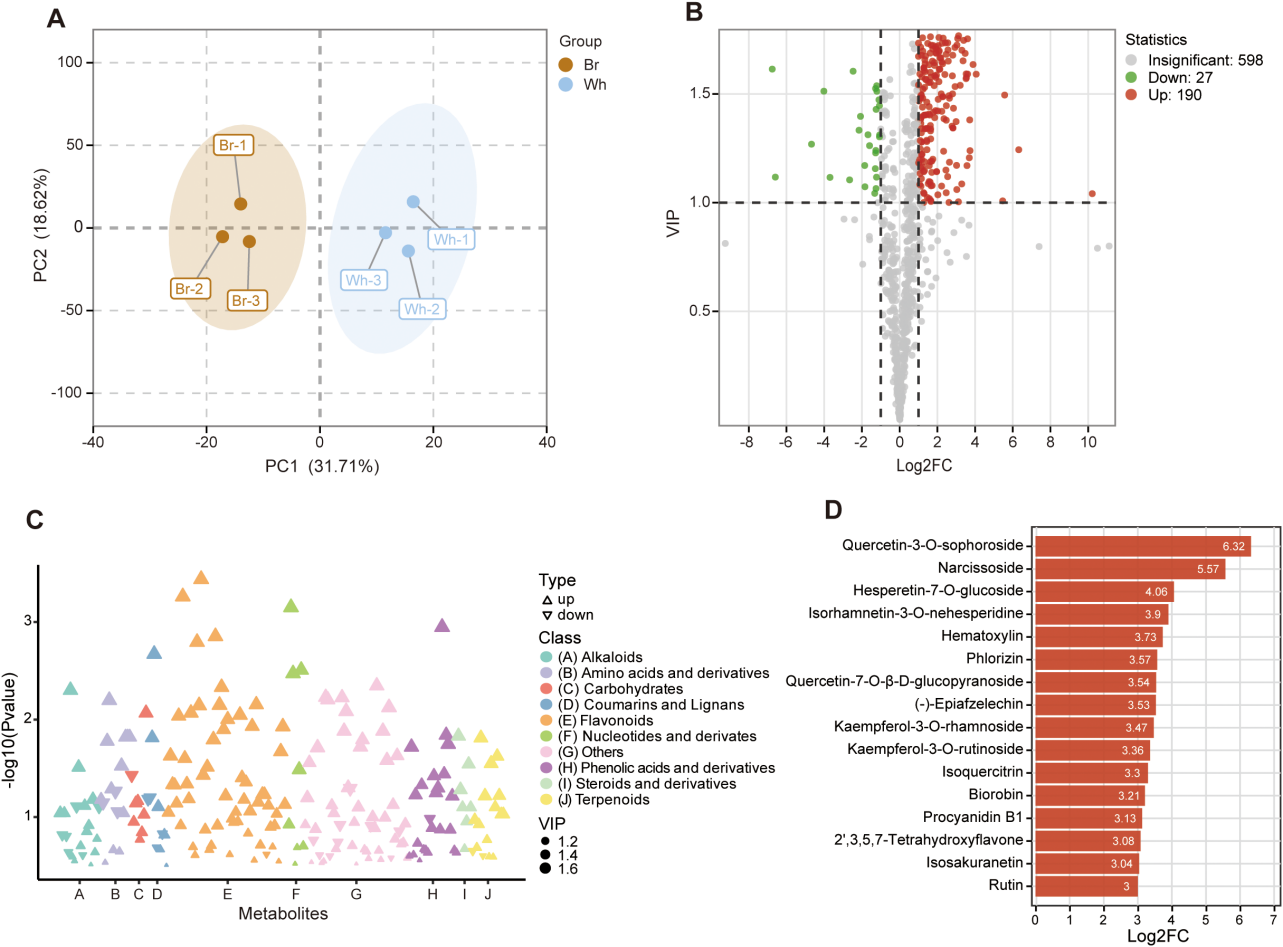
Significantly changed metabolites (SCMs) in yellow-brown *T. hemsleyanum* roots compared to whitish roots. **(A)** Principal component analysis of metabolite differences between the two tuberous roots. **(B)** Volcano plot of the metabolites between the two tuberous roots. **(C)** Classification of SCMs between the two tuberous roots. **(D)** Significantly changed flavonoids (SCFs) with the biggest fold change between the two tuberous roots.

The metabolic differences between whitish and yellow-brown roots were further characterized through a comprehensive metabolomic analysis. A total of 815 metabolites were identified in the tuberous roots of *T. hemsleyanum*, including 129 flavonoids, 88 alkaloids, 74 terpenoids, 53 amino acids and derivatives, and 30 nucleotide and derivatives (**Supplementary Table S3**). The metabolite variability was further verified by the results of PCA, which showed that significantly different levels of metabolites in the whitish and yellow-brown roots (**Figure 4A**). Compared to the metabolites in whitish roots, we found 217 SCMs in yellow-brown roots, with 190 being up-regulated and 27 down-regulated (**Figure 4B**). We further analyzed the classification of SCMs and found that over a quarter of the metabolites were flavonoids (**Figure 4C**). Of the 58 significantly changed flavonoids (SCFs), 57 were enriched in the yellow-brown roots, such as Quercetin-3-*O*-sophoroside, Narcissoside, Hesperetin-7-*O*-glucoside, Isorhamnetin-3-*O*-nehesperidine, Hematoxylin, and Phlorizin, which also exhibited the greatest content difference between the two roots (**Figure 4D**; **Supplementary Table S4**). Only one flavonoid (Hydrangenol) was highly elevated in whitish roots (**Supplementary Table S4**). These results indicated a significant difference in the enrichment degree and metabolic pattern of flavonoid metabolites between yellow-brown and whitish roots, further revealing the specificity of metabolite composition of *T. hemsleyanum* roots with different colors.

### Different contents of active flavonoids in the two tuberous roots

**Figure 5:**
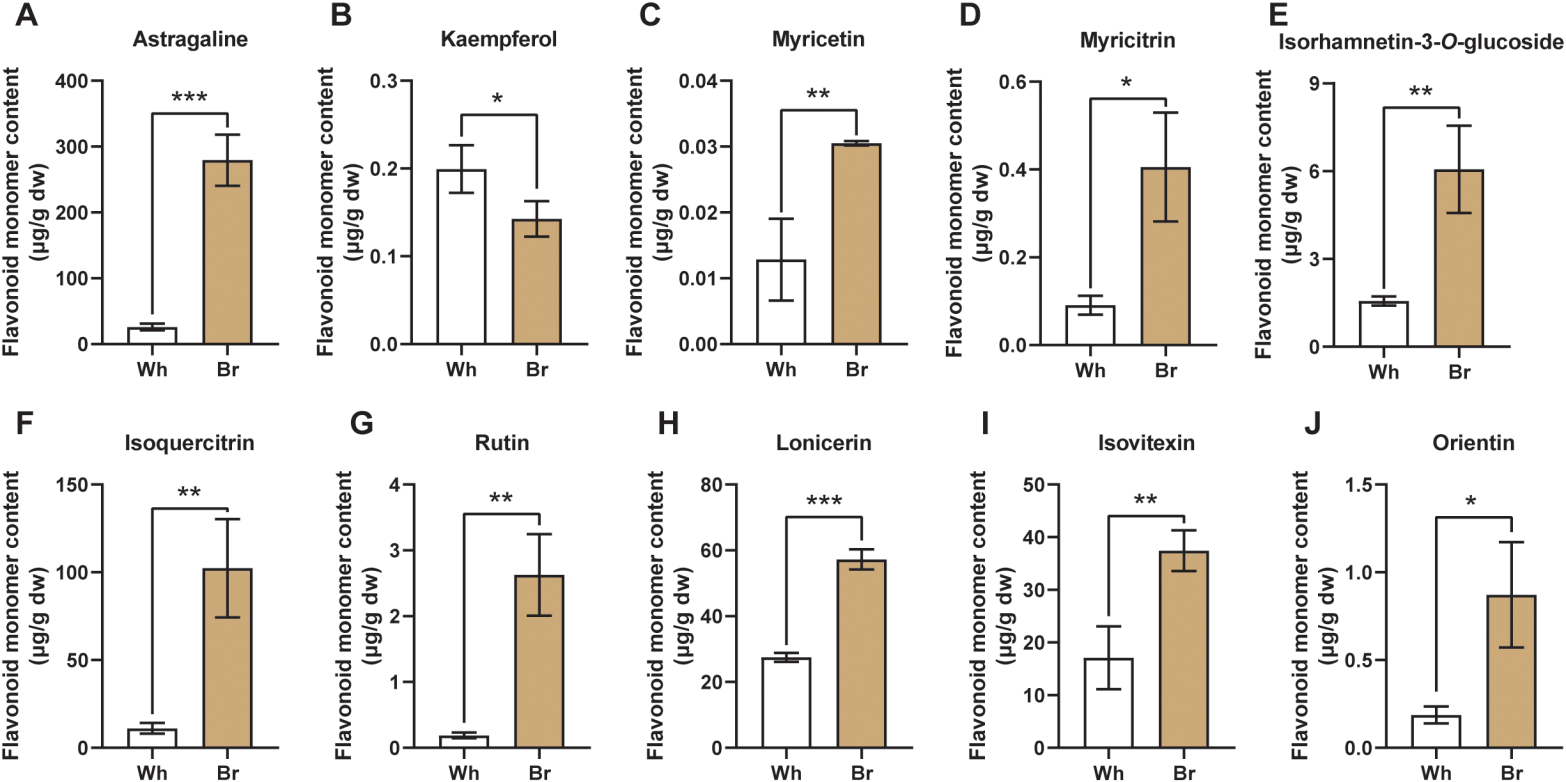
Flavonoid monomers with significant content differences between the two *T. hemsleyanum* tuberous roots. **(A–J)** The different contents of Astragaline, Kaempferol, Myricetin, Myricitrin, Isorhamnetin-3-*O*-glucoside, Isoquercitrin, Rutin, Lonicerin, Isovitexin, and Orientin. *, **, and *** indicate significant differences at *P* < 0.05, *P* < 0.01, and *P* < 0.001, respectively.

The differences in 16 major active flavonoids of *T. hemsleyanum* were explored through targeted metabolomic analysis. As shown in **Figure 5**, the contents of Astragaline, Kaempferol, Myricetin, Myricitrin, Isorhamnetin-3-*O*-glucoside, Isoquercitrin, Rutin, Lonicerin, Isovitexin, and Orientin are significantly different between the whitish and yellow-brown roots. Meanwhile, the contents of Isorhamnetin, Naringenin, Naringin, Hesperidin, Neohesperidin, and Calycosin-7-Oglucoside were similar between the two tuberous roots (**Supplementary Figure S1**). The content of Astragaline (Kaempferol-3-β-D-glucopyranoside) in the yellow-brown roots was about 279.35 μg/g, 10 times higher than that in the whitish roots, which was also higher than the other flavonoids (**Figure 5A**). In contrast, the kaempferol content in the whitish roots was 0.20 μg/g, slightly higher than that in the yellow-brown roots (**Figure 5B**). In terms of flavonols, the contents of Myricetin, Myricitrin, Isorhamnetin-3-*O*-glucoside, Isoquercitrin, and Rutin in the yellow-brown roots were 0.03, 0.41, 6.06, 102.32, and 2.63 μg/g, respectively, significantly higher than those in the whitish roots (**Figures 5C–G**. Furthermore, 3 flavone glycosides, i.e., Lonicerin, Isovitexin, and Orientin, were also significantly accumulated in the yellow-brown roots, reaching 52.23, 37.42, and 0.87 μg/g, respectively (**Figures 5H–J**). However, the content of flavonones and their glycosides, including Naringenin, Naringin, Hesperidin, and Neohesperidin, exhibited no significant differences between the two types of roots (**Supplementary Figure S1**). Considering the antioxidant assay results, it is clear that the material basis for the different medicinal activities of the two tuberous roots is flavonoids, especially flavonols and flavones.

### Differential expression of flavonoid biosynthesis-related genes in the two tuberous roots

To clarify the reasons behind the different flavonoid metabolites of the two types of tuberous roots, transcriptome sequencing and analysis were performed to find the key regulatory genes. About 33.23 Gb of clean data were obtained, and each sample had a Q30 value of > 93%, indicating that the derived data were of high quality (**Supplementary Table S5**). After the assembly, a total of 31326 unigenes were obtained from the transcriptome data, of which 75.38% could be annotated in at least one of the six databases.

**Figure 6:**
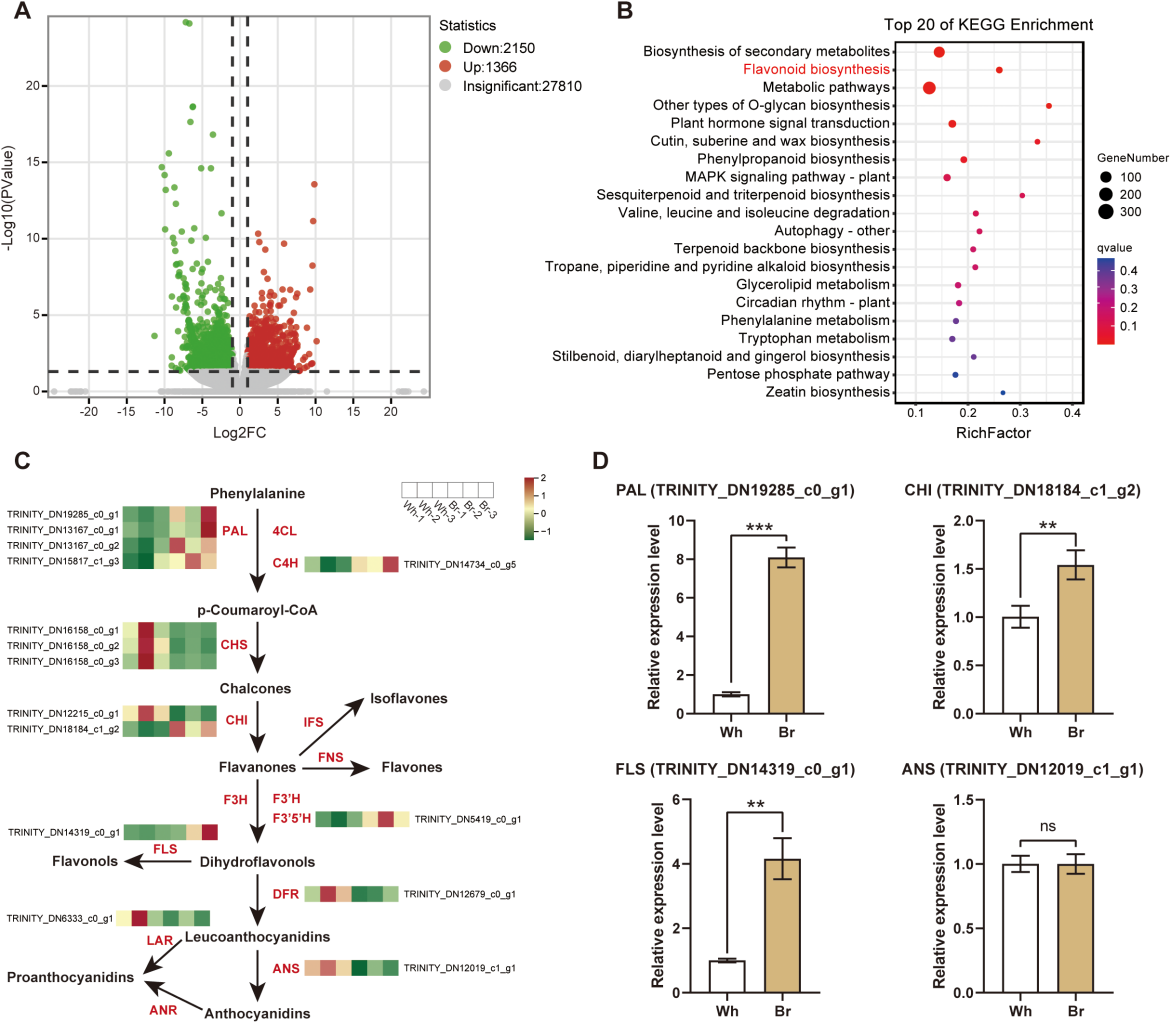
Differentially expressed genes (DEGs) in the yellow-brown *T. hemsleyanum* roots compared to the whitish roots. **(A)** Volcano plot of the gene comparison between the two types of roots. **(B)** KEGG enrichment of DEGs of the two tuberous roots. **(C)** Expression heatmap of structural genes of the flavonoids biosynthesis pathway. PAL, phenylalanine ammonia-lyase; 4CL, 4-coumaroyl-CoA ligase; C4H, cinnamate-4-hydroxylase; CHS, chalcone synthase; CHI, chalcone isomerase; IFS, isoflavone synthase; FNS, flavone synthase; F3H, flavanone 3-hydroxylase; F3’H, flavanone 3’-hydroxylase; F3’5’H, flavanone 3’,5’-hydroxylase; FLS, flavonol synthase; DFR, dihydroflavonol 4-reductase; LAR, leucoanthocyanidin reductase; ANS, anthocyanidin synthase; ANR, anthocyanidin reductase. **(D)** The relative expression levels of *PAL*, *CHI*, *FLS*, and *ANS* genes analyzed by qPCR. *, **, and *** indicate significant differences at *P* < 0.05, *P* < 0.01, and *P* < 0.001, respectively, and ns indicates no significant difference.

We compared the expression levels of all genes in the two tuberous roots and found a total of 3516 DEGs; 1366 genes were up-regulated, and 2150 genes were down-regulated compared to the whitish roots (**Figure 6A**; **Supplementary Table S6**). Then, the DEGs were annotated into the KEGG database for enrichment analysis, and a large number of DEGs were enriched in the flavonoids biosynthesis pathway (**Figure 6B**), demonstrating that the different medicinal qualities of the two *T. hemsleyanum* roots are mainly due to differential flavonoids biosynthesis. DEGs involved in the flavonoid biosynthesis pathway mainly included *PAL*, *C4H*, *CHS*, *CHI*, *F3′5′H*, *FLS*, *DFR*, *LAR*, and *ANS* genes (**Figure 6C**). The unigenes annotated as *CHS*, *DFR*, *LAR*, and ANS genes had higher expression levels in the whitish roots, while *PAL*, *C4L*, *F3′5′H*, and *FLS* unigenes expressed at high levels in the yellow-brown roots. The RT-qPCR results also showed significantly higher expression levels of *PAL*, *CHI*, and *FLS* genes in the yellow-brown roots, whereas the *ANS* gene was expressed similarly in the two roots (**Figure 6D**). Overall, the biosynthesis of flavonoids, especially flavonols, was stronger in the yellow-brown roots.

### Screening of transcription factors regulating flavonoid biosynthesis in tuberous roots

**Figure 7:**
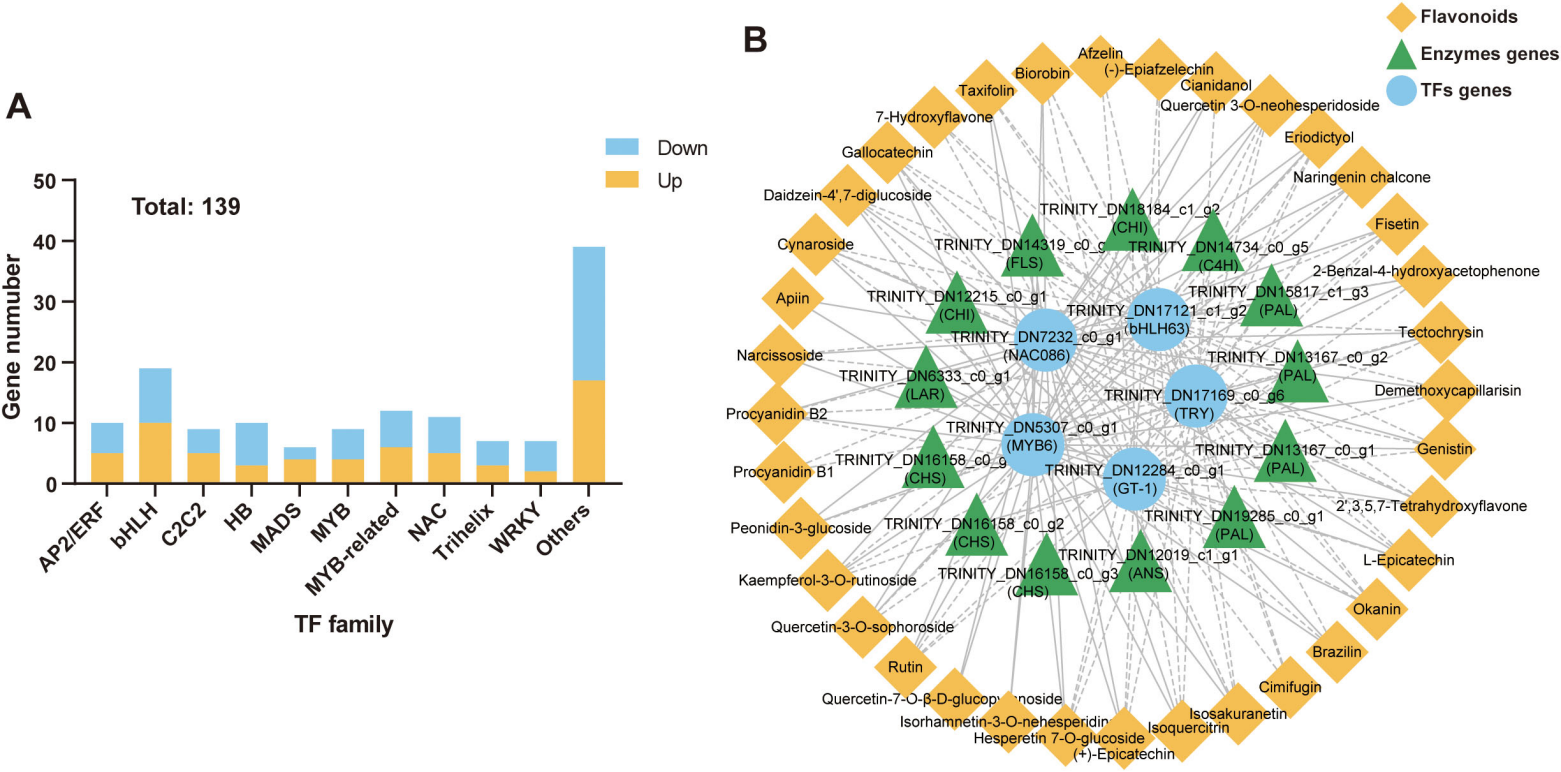
Candidate transcription factors regulating flavonoid biosynthesis in *T. hemsleyanum*. **(A)** Differentially expressed transcription factors genes (DETFs) in the yellow-brown roots compared to the whitish roots. **(B)** The Correlation network among SCFs, differentially expressed flavonoids biosynthesis genes (DEFGs), and differentially expressed transcription factor genes (DETFs).

Based on protein structural characteristics, the 139 DEGs (64 up-regulated and 75 down-regulated) were classified as transcription factor genes (**Figure 7A**), mainly including the bHLH, MYB-related, NAC, AP2/ERF, HB, and MYB families (**Supplementary Table S7**). To screen and mine the transcription factors regulating flavonoid biosynthesis in the tuberous roots of *T. hemsleyanum*, we performed a correlation analysis among the SCFs, differentially expressed flavonoid biosynthesis genes, and transcription factor genes. The results showed that *MYB6* (TRINITY_DN5307_c0_g1); *NAC086* (TRINITY_DN7232_c0_g1); *bHLH63* (TRINITY_DN17121_c1_g2); *GT-1* (TRINITY_DN12284_c0_g1); and *TRY* (TRINITY_DN17169_c0_g6) had the highest levels of associations with SCFs and DEFGs (**Figure 7B**; **Supplementary Table S8**). Among them, the *MYB6* and *NAC086* genes were co-expressed with *CHI* (TRINITY_DN18184_c1_g2) and *FLS* (TRINITY_DN14319_c0_g1) genes in the tuberous roots of *T. hemsleyanum*. However, *bHLH63*, *GT-1*, and *TRY* were significantly negatively correlated with these two genes and others, such as PAL genes (TRINITY_DN19285_c0_g1, TRINITY_DN13167_c0_g1, and TRINITY_DN13167_c0_g2). Besides, the expression levels of *MYB6* and *NAC086* were positively correlated with 31 flavonoids, including Isoquercitrin, Quercetin 3-*O*-neohesperidoside, Rutin, and Procyanidin B2, suggesting that MYB6 and NAC086 might be the transcription activators of flavonoids biosynthesis. In contrast, bHLH63, GT - 1, and TRY factors had an inhibitory effect on flavonoid biosynthesis.

### Functional analysis of the ThMYB6 factor

**Figure 8:**
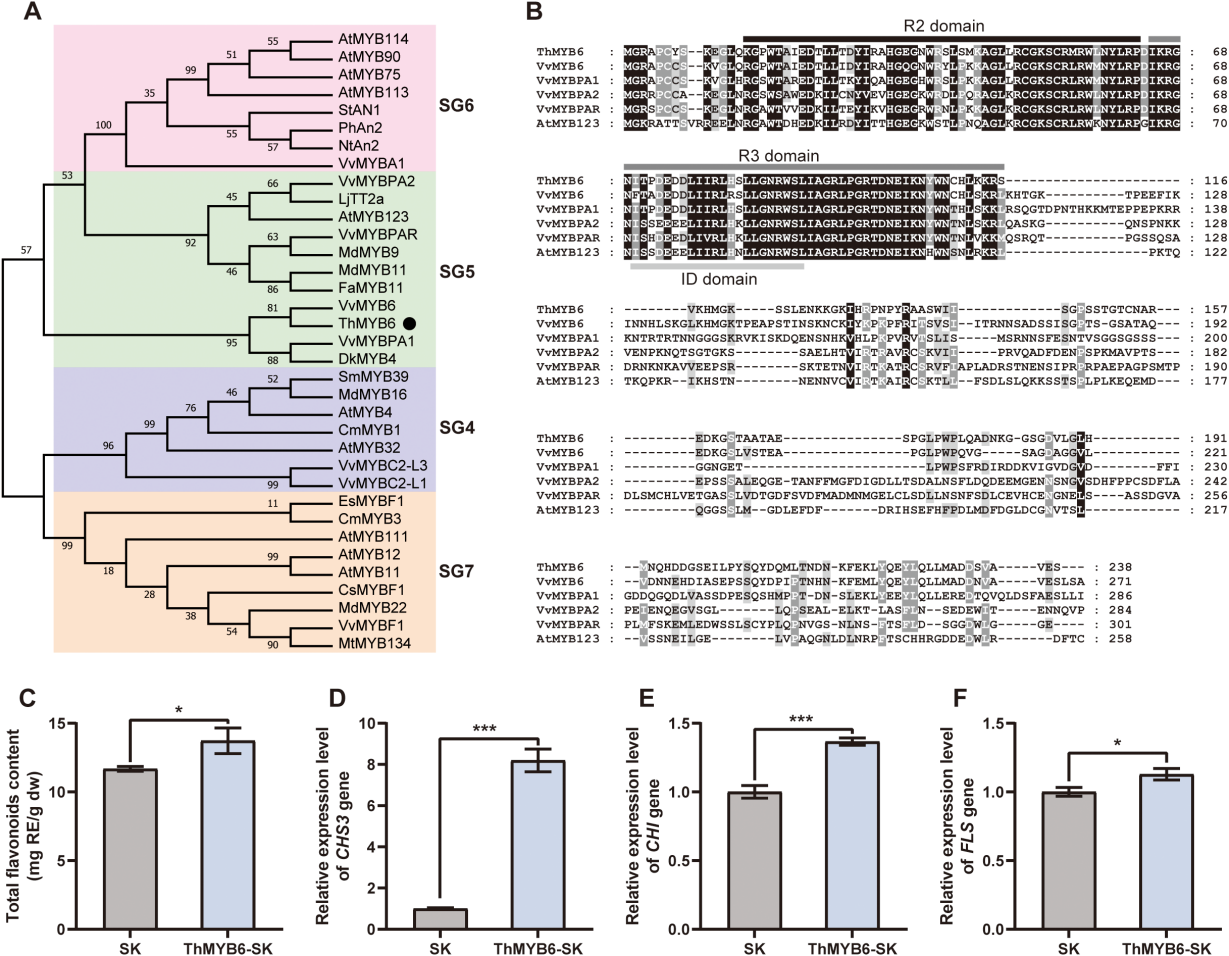
Sequence and functional analysis of ThMYB6 factor. **(A)** The phylogenetic tree of R2R3 MYB factors. **(B)** The multiple sequence alignment maps, where the conserved R2R3 domain and ID domain were indicated with the horizontal line. The full names of the species are as follows: At, *Arabidopsis thaliana*; Cm, *Chrysanthemum morifolium*; Cs, *Citrus sinensis*; Dk, *Diospyros kaki*; Es, *Epimedium sagittatum*; Fa, *Fragaria ananassa*; Lj, *Lotus japonicus*; Md, *Malus domestica*; Nt, *Nicotiana tabacum*; Sm, *Salvia miltiorrhiza*; St, *Solanum tuberosum*; Ph, *Petunia hybrida*; Vv, *Vitis vinifera*. **(C)** Total flavonoids content in the leaves of transient expression. **(D–F)** The relative expression levels of *CHS3*, *CHI*, and *FLS* genes in the leaves of transient expression.

As MYB factors have been recognized as the primary regulators of flavonoid biosynthesis, we analyzed the functions of MYB6. We cloned a 714 bp gene based on transcriptome data and named it ThMYB6. The ThMYB6 gene was highly similar to the VvMYB6 gene (Genbank ID: MN125488), which positively regulated flavonoid biosynthesis in Vitis vinifera (Zhu et al., 2021). The functions of ThMYB6 were analyzed via phylogenetic analysis between ThMYB6 and other R2R3 MYB factors. In the phylogenetic tree, ThMYB6 was grouped with DkMYB4 and VvMYBPA1, falling into the R2R3 MYB subgroup 5 (**Figure 8A**). Members of SG5 are believed to promote proanthocyanidin biosynthesis. Further MYB protein sequence analysis revealed that it indeed possesses a typical R2R3 MYB domain (**Figure 8B**). Besides, an ID domain ([D/E]Lx2[R/K]x3Lx6Lx3R) was found, which was involved in the interaction with bHLH proteins and was present in the SG5 R2R3 MYB proteins. Therefore, ThMYB6 was predicted to regulate the biosynthesis of flavonoids in *T. hemsleyanum*.

The functional characterization of ThMYB6 was validated by transient overexpression assays in the leaves of *T. hemsleyanum*. Quantitative analysis showed that the total flavonoid content in leaves overexpressing ThMYB6 was 20% higher than that in the control plants (**Figure 8C**). Furthermore, transcript profiling demonstrated significant up-regulation of multiple structural genes in the flavonoids biosynthesis pathway, with particularly notable enhancement of *CHS3* gene expression (**Figures 8D–F**). The *CHS*3 transcript level in transgenic leaves reached 8-fold higher than that observed in the control group (**Figure 8D**), indicating a strong regulatory effect of ThMYB6 on this pivotal enzyme in flavonoid production.

## Discussion

Color is an important characteristic of plants, which can be attributed to different kinds and contents of metabolites. In authentic Chinese medicine, color is regarded as an excellent indicator of high-quality herbs (Yuan and Huang, 2020), as exemplified by the red roots of *Salvia miltiorrhiza* and the yellow roots of *Scutellaria baicalensis*. This research compared the phenotypes and antioxidant activities of the two *T. hemsleyanum* types, and the results linked the antioxidant activities to their colors. The high flavonoid content of yellow-brown *T. hemsleyanum* roots was the material basis for strong antioxidant capacities. Phenolic compounds (total flavonoids, Quercetin, and Rutin) are the main reason for the antioxidant capacity of the Verbascum flower extracts (Amini et al., 2022). Both red and black rice have significant antioxidant capacities compared to white rice, attributed to catechins and quercetin, respectively (Chen et al., 2022b). Myricetin is a natural antioxidant flavonoid with various biological activities (Xia et al., 2020). Myricitrin, the predominant flavonoid in *Syzygium antisepticum* leaves, exhibits considerable DPPH scavenging activity (Mangmool et al., 2021). Among six compounds isolated from sea buckthorn leaves, quercetin 3-*O*-β-d-Glucopyranoside has the highest free radical scavenging activity (Kim et al., 2011). In addition, flavonoids were identified as the main contributors to the color difference between the two tuberous roots of *T. hemsleyanum*. Moreover, transcriptomic and metabolomic analyses offer significant advantages in uncovering the biosynthetic mechanisms of key metabolic pathways (Gao et al., 2023). Based on metabolomic analysis, 129 flavonoids were found in the *T. hemsleyanum* roots. Among them, 57 SCFs were higher in the yellow-brown tuberous roots. We performed a color query of the 57 SCFs by Chemical Book (https://www.chemicalbook.com/) and found that most of them were yellow or pale-yellow, including Narcissoside, Isoquercitrin, and L-Epicatechin. Flavonoids are stored as water-soluble pigments in the vesicles of plant cells, including anthocyanins (red, orange, blue, and purple pigments), chalcones and aurones (yellow pigments), and flavonols and flavones (white and pale-yellow pigments), which confer a wide range of colors to plants (Dong and Lin, 2021; Grotewold, 2006). Research has found that isoquercitrin is one of the seven phenolic pigment monomers responsible for the colors of different lily bulbs (Liang et al., 2022). Proanthocyanidins are the most important differentially accumulated metabolites contributing to the difference between yellow and black seeds of *Brassica rapa* (Zhao et al., 2022). The yellow-brown seeds of *Perilla frutescens* have also been found to contain higher levels of flavonoids (Dossou et al., 2023). The findings of this study are similar to those in the literature.

This study combined metabolomic and transcriptomic analyses to investigate the molecular mechanisms for the different qualities and colors of two types of *T. hemsleyanum* roots. The results identified 3516 DEGs between the two tuberous roots, and a large number of DEGs were enriched in the flavonoid biosynthesis pathway. The different qualities and colors of the two roots were due to different levels of flavonoid biosynthesis at the molecular level. Besides, *PAL*, *C4L*, *F3’5’H*, and *FLS* genes were found highly expressed in yellow-brown roots, suggesting them as the key genes of flavonoid biosynthesis in *T. hemsleyanum* roots. Research also showed that the transcript abundance of *PAL, CHI, DFR, LDOX*, and *UFGT* gene expression was increased in the dark blue type *Myrtus communis* L. compared to the white type, with a strong positive correlation between the changes in gene expression and anthocyanin accumulation (Medda et al., 2021). In *Rhododendron pulchrum*, the up-regulation of *F3′H* and *F3′5′H* and the down-regulation of *4CL, DFR, ANS*, and *GT* have been associated with the pink coloration. In the meantime, pigment accumulation can be inhibited by the low expression of *F3′5′H, DFR*, and *GT*, leading to a white coloration (Xia et al., 2022). The expression of *CmFNS* and *CmFLS* was found to be negatively correlated with the red petal color of *Chrysanthemum morifolium*, which was attributed to anthocyanins (Wang et al., 2021). Furthermore, flavonoid biosynthesis is often tightly linked to environmental factors. For example, under varying light regimes, inflorescences of *Chrysanthemum morifolium* display distinct coloration. Shading significantly downregulates floral expression of *CmPAL*, *CmCHS1/2*, *CmF3’H*, and *CmFNS*, reduces flavonoid levels, and thus leads to white coloration (Lu et al., 2024). In the present study, plant materials collected from the same cultivation site displayed distinct phenotypic variations. We therefore hypothesize that these differences result from environmental factors, such as variations in light exposure. This hypothesis is supported by the observation that *T. hemsleyanum* is typically cultivated beneath forest canopies, where light regimes differ significantly beneath different tree species.

Certain transcription factor genes have been predicted to regulate the flavonoids biosynthesis in *T. hemsleyanum* roots, such as the MYB, bHLH, and NAC families. In *Arabidopsis thaliana*, many MYB factors from different subgroups can function to promote or repress the expression of flavonoid biosynthesis genes. For instance, AtMYB11, AtMYB12, and AtMYB111 are transcription activators of flavonol biosynthesis (Stracke et al., 2007, Stracke et al., 2010), while AtMYBL2 acts as a transcription repressor to regulate anthocyanin biosynthesis (Matsui et al., 2008). Meanwhile, bHLH could bind MYB and WD40 factors to form protein complexes regulating the biosynthesis of flavonoids, such as anthocyanins and proanthocyanidins (Ramsay and Glover, 2005; Jiang et al., 2023). In *Malus domestica*, *MdNAC42* and *MdNAC52* have been proven to participate in flavonoid biosynthesis by interacting with *MdMYB* factors (Sun et al., 2019; Zhang et al., 2020). This study found that the *MYB6* gene co-expressed with *CHI* and *FLS* genes, thus closely related to flavonoid biosynthesis and accumulation in *T. hemsleyanum*. According to the annotation and sequence alignment results, ThMYB6 is highly similar to the VvMYB6 factor, which has been reported to promote the anthocyanin biosynthesis of grapevine (Zhu et al., 2020). This study also found that ThMYB6 can increase the content of flavonoids in *T. hemsleyanum* leaves by promoting the expression of *CHS3*, *CHI*, and *FLS* genes. Therefore, ThMYB6 might be a positive regulator of flavonoid biosynthesis in the tuberous roots of *T. hemsleyanum*.

## Conclusion

In summary, this study reported the discovery of a *T. hemsleyanum* type exhibiting a yellow-brown root cross-section color and investigated the underlying biochemical mechanisms. Metabolomic profiling revealed 129 differentially accumulated flavonoids between the two color morphotypes. Integrated metabolomic and transcriptomic analyses identified several key metabolites, genes, and transcription factors associated with the cross-section color of *T. hemsleyanum*. Notably, ThMYB6 was functionally characterized as a pivotal regulator driving elevated flavonoid accumulation in the yellow-brown tuberous roots. The findings established flavonoid biosynthesis as the primary determinant of phytochemical variation between the two types of tuberous roots. This work provides novel insights into flavonoid metabolism in the *T. hemsleyanum* and establishes a molecular foundation for quality optimization and sustainable utilization of this medicinally valuable species.

## Data Availability

The datasets presented in this study can be found in online repositories. The RNA-seq data have been deposited to the NCBI and are publicly available under accession number PRJNA1306786.

## Supplementary material

Supplementary Figure 1 Flavonoid monomers without significant content differences between the two *T. hemsleyanum* tuberous roots. **(A-F)** The contents of Isorhamnetin, Naringenin, Naringin, Hesperidin, Neohesperidin, and Calycosin-7-*O*-glucoside, where ns indicates no significant difference.
