# Supplementary figures and images for: Multi-omics analysis reveals ThMYB6 regulation of flavonoid biosynthesis in differently colored tuberous roots of Tetrastigma hemsleyanum
Source: Front Plant Sci. 2025 Sep 8;16:1642835. doi: 10.3389/fpls.2025.1642835 (PMC12450873; doi:10.3389/fpls.2025.1642835)

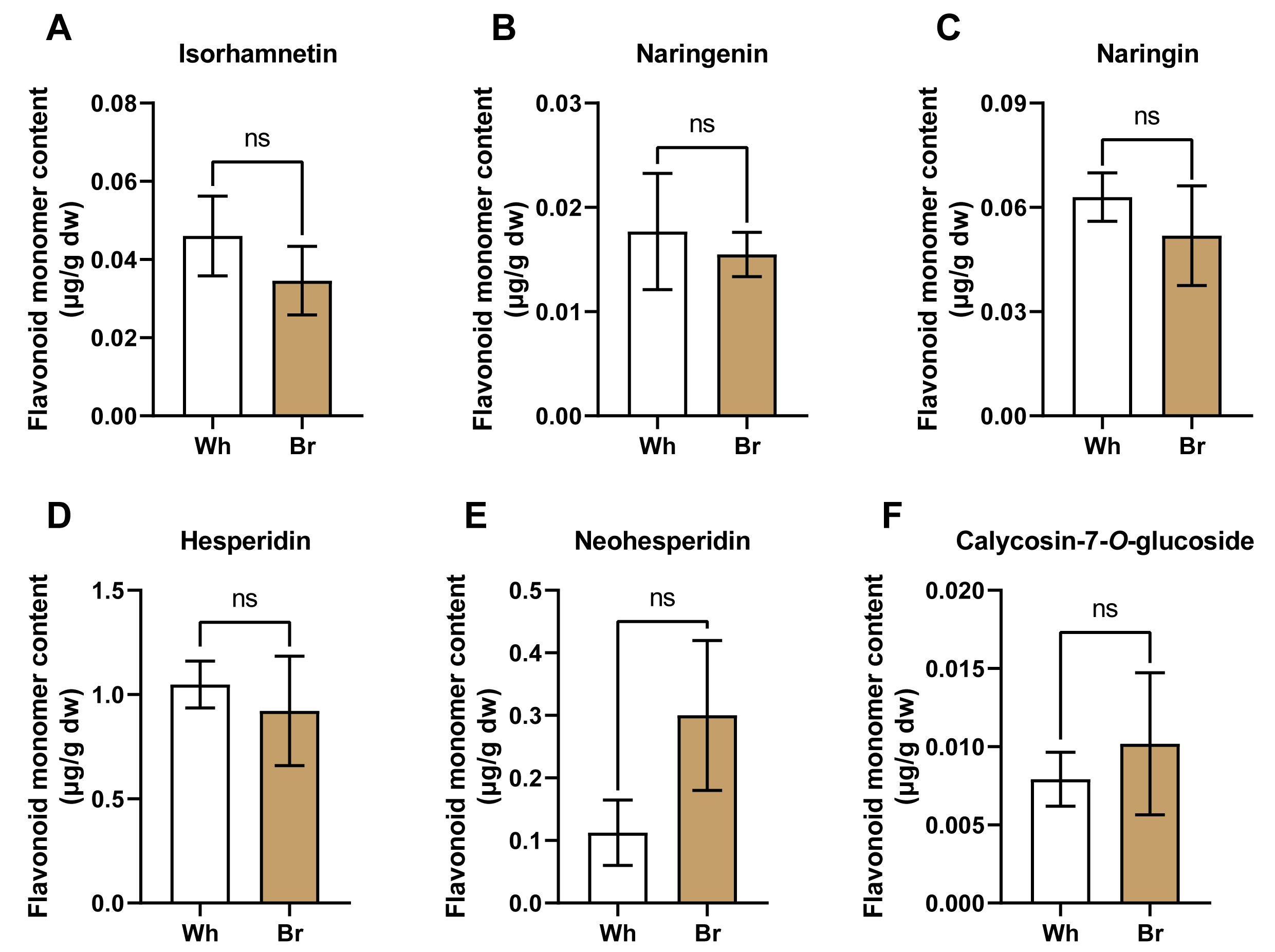

Supplement: Supplementary Figure 1 — Flavonoid monomers without significant content differences between the two T. hemsleyanum tuberous roots. (A-F) The contents of Isorhamnetin, Naringenin, Naringin, Hesperidin, Neohesperidin, and Calycosin-7-O-glucoside, where ns indicates no significant difference. [file Image1.tif]
